# New Inventions

**Published:** 1896-04

**Authors:** 


### NEW INVENTIONS.

A rubber horsehoe-pad, arranged to cover the rear part of hoof, and used with a three-quarter shoe, which is recessed into depressions in the rubber pads.

A nailless horseshoe, of usual form of construction, with metallic straps welded to outside of rear portion of the wings of the shoe, and connected with metallic braces to forward part of shoes, with a clamping-device at the ends of said straps, the latter passing through each other.

A horse-controller, consisting of a rod of metal formed with a pair of bib strap-loops, a wide U-shaped section extending forward and joining the strap-loops, and a section for adjusting extending backward and adjusted to the throat-latch.

A horse-tail tie, consisting of two lengths of coiled wire, parallel with one another, and fastened to a metal plate at one end, so arranged to receive the other end, which is made adjustable.

A device for training horse's manes, consisting of two wooden bars, with rubber facing secured by clamps, and so attached to the mane.

A toe-calk, with a vertical groove on one face from top to bottom, adapted to receive and frictionally hold a nail, securing the calk firmly during the welding to the shoe.

A horse-blanket, with adjustable breast-stays and cross-bands around the body fastened by hooks and rings.

Method for preparing a dried form of meat-extract and malt by desiccation after maceration.

An improved means of attaching snap-hooks to straps through the medium of a metal plate.

An improved horseshoe, consisting of a body of soft metal, faced for the wearing surface with a strip, and calkings of hard metal.

A powder-atomizer, consisting of a rubber bulb and a discharge-nozzle connected with an agitator-tube for the powder.

A wire tail-guard for horses.

A currycomb, consisting of a metal framework toothed on the bottom, a series of parallel bars toothed on both edges, so arranged as to revolve in the body framework.

A horseshoe, having a gripping border projecting below the main portion of ground surface of shoe.

Ligature-holder and receptacle, consisting of a glass bottle or jar with capped mouth, and a central spindle for winding upon the thread, so arranged in the jar as to be free of contact of the sides, and the spindle revolving, allowing the removal of silk or thread as required, the central spindle or cylinder being fastened by movable disks.

A device for removing feathers from fowls, the gripper revolving and opening and closing for the purpose intended.

An animal-releasing device, consisting of a slotted base-block, a locking-bar working through said block, a locking-bolt working in the base-block.

An automatic watering-trough.

A self-opening pocket-knife, by a pivoted, spring-actuated blade.

A horse-collar, consisting of a stuffed roll with metal casing, and a draft-bearing-pad separated therefrom so as to form a hame-crease; metallic casing covering partially the external surface of the pad, the portion bearing against the animal covered with a leather casing joined at its edges to the metal casing.

A knee boot-holder, consisting of a non-elastic shoulder-strap, elastic and flexible suspending loops at ends of shoulder-strap, rubber holding parts passing loosely through the loops and provided with button tabs at the ends.

A combination bridle-bit, the mouthpiece having a central arch; a spindle extending horizontally through said mouthpiece and having an arch parallel with the arch thereof and normally resting against the cheekpieces secured to the ends of the spindles; coiled springs between ends of spindle and secured to cheek- and mouthpieces and to the driving-reins, the mouthpiece and spindle turning in opposite directions.

A horseshoeing apparatus, consisting of a frame support for a series of levers and attachments for the limbs, by which the latter are secured in suitable positions to enable the shoer to adjust more readily the shoes.

Veterinary obstetrical forceps, one of the bars having at its end a fenestrated blade, the other an upwardly curved point; the other bar an upwardly projecting ball to coact with the blade, the other end a wide curved face.

